# Dynamics of spatiotemporal line defects and chaos control in complex excitable systems

**DOI:** 10.1038/s41598-017-08011-z

**Published:** 2017-08-10

**Authors:** Marcel Hörning, François Blanchard, Akihiro Isomura, Kenichi Yoshikawa

**Affiliations:** 1 Institute of Biomaterials and Biomolecular Systems (IBBS), University of Stuttgart, Stuttgart, 70569 Germany; 2 Institute for Integrated Cell-Material Sciences, Kyoto University, Kyoto, 606-8501 Japan; 3 Department of Electrical Engineering, École de Technologie Supérieure, 1100 Notre-Dame Ouest, Montréal, Québec, H3C 1K3 Canada; 4 Institute for Frontier Life and Medical Sciences, Kyoto University, Kyoto, 606-8507 Japan; 5 Japan Science and Technology Agency, PRESTO, Saitama, 332-0012 Japan; 6 Faculty of Life and Medical Sciences, Doshisha University, Kyotanabe, Kyoto, 610-0394 Japan

## Abstract

Spatiotemporal pattern formation governs dynamics and functions in various biological systems. In the heart, excitable waves can form complex oscillatory and chaotic patterns even at an abnormally higher frequency than normal heart beats, which increase the risk of fatal heart conditions by inhibiting normal blood circulation. Previous studies suggested that line defects (nodal lines) play a critical role in stabilizing those undesirable patterns. However, it remains unknown if the line defects are static or dynamically changing structures in heart tissue. Through *in vitro* experiments of heart tissue observation, we reveal the spatiotemporal dynamics of line defects in rotating spiral waves. We combined a novel signaling over-sampling technique with a multi-dimensional Fourier analysis, showing that line defects can translate, merge, collapse and form stable singularities with even and odd parity while maintaining a stable oscillation of the spiral wave in the tissue. These findings provide insights into a broad class of complex periodic systems, with particular impact to the control and understanding of heart diseases.

## Introduction

Spatiotemporal dynamics in excitable media appear in a wide and diverse range of systems^1–3^. In biological systems in particular, these dynamics have important regulatory functions. An example is PIP3-lipid dynamics in the membranes of Dictyostelium cells regulating cell migration^4, 5^. Other examples include electrophysiological waves in the cerebral neocortex^6^ and mammalian hearts, where the latter maintain the contractility and cardiovascular blood circulation^7^. Disturbances in these excitable patterns can cause severe, and even fatal, impacts on the organism. In the heart, perturbations can induce wave breakups that lead to spiral waves (arrhythmia)^8^, which can further evolve into life-threatening dynamics with spatiotemporally chaotic waves (fibrillation)^9^. Spiral waves occur as freely meandering or obstacle-anchored rotors that are even more difficult to terminate, thus increasing the risk of fibrillation^10–13^.

Another risk factor to inducing fibrillation is beat-to-beat alternans in the action potential duration (APD)^14^, which promotes an increase in the variations of the refractory period and conduction velocity (CV)^15–18^. Experimentally, a combination of drugs (Bay K 8644 and isoproterenol) or low temperatures (22–24°C)^19, 20^ can induce alternans with the steep restitution properties. The alternation of short and long APDs (period-2 oscillation) is also similarly observed in the intracellular calcium dynamics^21^. Generally, alternans is initiated either by a steep APD restitution of the membrane potential or a steep function governing calcium ion (Ca^2+^) release into the intracellular space^16^, which leads to the alternation of the calcium transients governed by a plateau voltage of the action potential^22, 23^. However, functional links between global alternans-patterns and local calcium dynamics remain to be determined.

**Figure 1:**
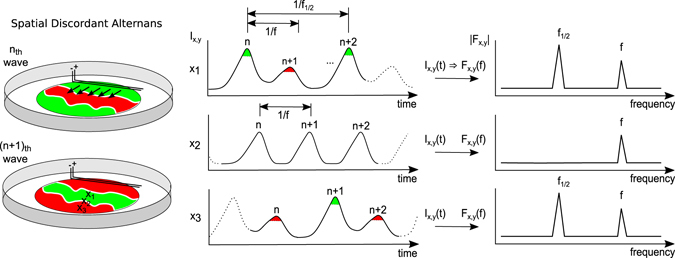
Schematic illustration of spatially discordant alternans in high-frequency oscillatory cardiac tissue. The three time series illustrate the temporal intracellular calcium release at three spatially different positions. Positions x_1_ and x_3_ are spatially separated by the nodal line (x_2_). The black arrows illustrate the direction of wave propagation originating from the electrode. The three frequency spectra on the right side show the respective amplitudes |*F*_*x*,*y*_(*f* )|. In case of period-1 oscillations (nodal lines, x_2_) no frequency- peak at *f*_1/2_ is detected and *f* is the rotational frequency of the spiral wave. Thus, a spatial amplitude map at *f*_1/2_ will reveal the nodal lines as low amplitude (low energy) bands in the tissue. (For a more detailed example that compares normal wave conduction, spatially discordant and spatially concordant alternans, see the supplemental material, Fig. S1).

Calcium alternans occurs in two forms other than normal (period-1) conduction in periodically oscillating tissue, namely spatially concordant alternans (SCA) and spatially discordant alternans (SDA). SCA is observed when the APD alternates in phase throughout the entire tissue^21^, and SDA occurs when the APD alternates out of phase in different regions^19^ (Fig. 1; also see the supplemental material for a schematic comparison, Fig. S1). The emergence of SDA was initially explained by the steep slope of the APD restitution curve^24, 25^ that is defined by the correlation between APD and diastolic interval (DI)^26^. The later studies showed that SDA is trigged by dynamic instabilities in the intracellular Ca^2+^ release from the sarcoplasmic reticulum (SR) in single cardiomyocytes^19, 27–29^. This is caused by the alternation of SR Ca^2+^ content and a steep relationship between SR Ca^2+^ content and its release. A larger calcium transient amplitude (CTA), the relative amplitude of each calcium oscillation, results from a higher SR Ca^2+^ content and vice versa^28, 30^. Whether APD and CTA alternate in or out of phase depends on the slope of CV restitution, i.e., the CV vs. DI relationship that is caused by the incomplete recovery (short DIs) from the inactivation of the fast sodium current causing slowing of CV^31^. SDA can only form independently of the CV restitution when CTA and APD are electromechanically out of phase, i.e., a large CTA corresponds to a short APD and vice versa^32^. In either case, the out-of-phase regions of SDA in the tissue are separated by nodal lines (NL), in which no alternans is present (see location *x*_2_ in Fig. 1)^19, 20, 33, 34^. A comprehensive and detailed review of SDA and NL in cardiac tissue from the perspective of bifurcation theory is presented by Karagueuzian and coworkers^31^. There are additional factors reviewed, such as tissue heterogeneities, cellular coupling and anatomic barriers, that can potentially influence the development of SDA.

Although many studies have investigated SDA and NL, the stability and dynamics of NL in heart tissue are still poorly understood. This is partially due to the lack of sophisticated imaging and analysis methods for extracting relevant information from the low and noisy signals in observations. Here, we attempt to solve this problem using a combination of spatiotemporal analysis techniques, i.e., an oversampling image processing technique^34^ and multidimensional Fourier analysis of quasi-steady-state dynamics. Using these tools we show that complex structures of NL can develop in freely rotating and obstacle-anchored spiral waves and that the NL exhibit various pattern formations, such as translation, merging, collapsing and formation of stable singularities with even and odd parities. Though a rich variety of constantly changing NL patterns has been observed in the cytosolic calcium signaling, the position of the spiral wave was unaffected except for a slow linear decline in the oscillation frequency. We found that with lower frequencies, the number of NL decreases, and an odd parity of NL crossings is linked to the formation and stability of spiral wave tips. Newly formed odd NL crossings cause the formation of spiral waves and are therefore a potential cause of chaotic-like wave formation in cardiac tissue, such as fibrillation.

## Materials and Methods

The protocol used for primary cardiomyocyte culture and electrical stimulation has been documented previously^21, 35, 36^.

### Cell culture

Hearts isolated from neonatal 2-day-old Wistar rats were minced and enzymatically digested using collagenase (Type-1, Wako). The isolated cells were collected by centrifugation and preplated for 1 h. The cells were plated on 22-mm-diameter glass coverslips coated with human fibronectin (12 g/ml, Gibco) at a cardiac cell density of 2.63 × 10^3^ cells/mm^2^. Cell constructs were incubated in Dulbecco’s Modified Eagle’s Medium (Gibco) with 10% fetal bovine serum (Gibco) and 1% penicillin streptomycin (Sigma) for 24 h under humidified conditions at 37 °C and 5% CO_2_. The medium was then replaced with a contraction medium, Minimum Essential Medium (Gibco) with 10% calf serum (Gibco), 1% penicillin streptomycin, and 1% cytosine arabino-furanoside (ARA-C, Sigma). The latter prevents overproliferation of contaminating nonmyocytes^37^ and thus stabilizes cardiac conduction^38^.

### Obstacle handling

The round obstacles (1.5 and 2.0 mm in diameter) were manufactured from polydimethylsiloxane (PDMS) on a silicon waver and placed on the coverslip before coating with fibronectin as documented previously^39^. The obstacle was removed after 24 hours of cell culturing when the medium was replaced by the Minimum Essential Medium (see Cell culture).

### Calcium visualization

Observations were performed four days after plating. Tissue was labeled with the Ca^2+^-sensitive fluorescent green indicator Fluo-8 (Fluo-8 AM; ABD Bioquest, Sunnyvale, CA, 1:11). Briefly, 50 *μ*g Fluo-8 was diluted in 45.6 *μ*/L of high-quality, anhydrous dimethylsulfoxide (DMSO). Aliquots (each 4 *μ*/L) of the indicator stock solution were stored until use in the dark at −20 °C. The DMSO stock solution was diluted to 1/10 (v/v) with Tyrode solution (Sigma) and 200 *μ*/L was added to the cells for 1 hour. The medium was washed and replaced with fresh Tyrode solution at room temperature (22–24° C) for the noninvasive promotion of SDA in the tissue cultures^19, 20^. The tissues were studied within 1 h after loading.

### Time lapse and image acquisition

Fluorescence was observed using an inverted microscope (IX-70; Olympus, Tokyo Japan) with a ×1.25 magnification objective lens (PLAPON, N.A. = 0.04; Olympus, Tokyo Japan) in combination with a ×0.35 intermediate lens. Raw images were obtained using an EMCCD camera (iXon DV887ECS-UVB; Andor) at a 14-bit resolution of 128 × 50 pixels with 200 frames/sec and 128 × 128 pixels with 119 frames/sec after 4 × 4 binning with an effective spatial resolution of 180 *μ*m/px.

### Electrical stimulation

For inducing spiral waves to the cardiac tissue electrical stimulation of 6 V was applied with 10-msec bipolar pulses delivered through 1-mm-spaced platinum electrodes on the edge of the sample and a second locally confined bipolar electrode close to the sample without physical contact with the tissue. The time-delayed secondary electrical stimulus was applied close to the obstacle in the vulnerable window of the initially induced propagating wave to induce an obstacle-bound or freely rotating spiral wave^35^ (see supplemental material, Fig. S3).

### Data analysis

Data were analyzed by MATLAB (R2016a; The MathWorks, Natick, MA) with custom routines.

### Oscillation stacking

The oscillation stacking procedure was adapted from the framework proposed by Uzelac and Fenton^34^. Briefly, the Ca^2+^-oscillation time series were recorded with 200 frames/sec for 10 sec. This corresponds to ca. 50 to 100 oscillations depending on the spiral wave frequency. The intensity minima of the oscillation were detected and computed to represent an average Ca^2+^-oscillation per time series. An average oscillation contains one larger and one smaller amplitude, thus in case of a period-2 oscillation only every second minimum was detected. To ensure that the dynamics do not change significantly within a single record, the spiral rotational frequency *f* is confirmed to be constant during the first and latter half of the time-series using Fourier transformation. That means that the change in frequency is in order of the temporal resolution of the record.

### Calcium signaling analysis

The magnitude of alternans in intracellular calcium signaling (CA-ALTM) is calculated as

1 $${\rm{CA}} \mbox{-} {\rm{ALTM}}=1-\frac{{{\rm{CTA}}}_{Small}}{{{\rm{CTA}}}_{Large}},$$

where CTA_*Large*_ is obtained from the larger CTA_*n*_ and CTA_*Small*_ is the subsequently occurring smaller CTA_*n+*1_^40–43^. The difference in the cycle length is calculated using the following formula

2 $$\begin{array}{rcl}{\rm{\Delta }}{\rm{CT}} & = & {\rm{\Delta }}{\rm{CTD}}+{\rm{\Delta }}{\rm{CTI}}\\  & = & | {{\rm{CTD}}}_{n}-{{\rm{CTD}}}_{n+1}| +| {{\rm{CTI}}}_{n+1}-{{\rm{CTI}}}_{n}| \end{array}$$

where the differences between the longer and shorter calcium transients are calculated from the CTD and CTI at an intensity level of 50%^21^.

### Fourier transformation imaging

Fluorescence optical mapping recordings were analyzed by Fourier transformation. The intensity sequences at each pixel of the images, *I*_*x*,*y*_(*t*), are transformed to the mathematically complex Fourier space, *F*_*x*,*y*_(*f*), as a function of the frequency *f*. From *F*_*x*,*y*_(*f*), the amplitude |*F*_*x*,*y*_(*f*)| and the phase *arg*(*F*_*x*,*y*_(*f*)) information of the signal is extracted at each pixel and spatially recomposed to a Fourier image for each frequency. Fourier images at the frequencies that represent the electrophysiological dynamics aid in visualizing the underlying spatiotemporal dynamics (Fig. 1). For example, a SDA exhibiting spiral wave, that rotates with the frequency *f*, is visualized in the Fourier space at *f* that shows the phase of the spiral wave (*arg*(*F*_*x*,*y*_(*f*)). Furthermore, *f/*2 (≡*f*_1/2_) can be used to show the oscillation amplitude (|*F*(*f*_1/2_)|) in case of an underlying period-2 dynamic. There local period-1 (NL) and period-2 oscillations exhibit different amplitudes and thus aid to visualize NL.

### Ethics statement

This study was carried out in strict accordance with the guidelines for animal experimentation established by the Animal Research Committee, Kyoto University. The protocol was approved by the Animal Research Committee, Kyoto University (Permit Number H22023).

## Results

### Analysis of spatially discordant alternans

**Figure 2:**
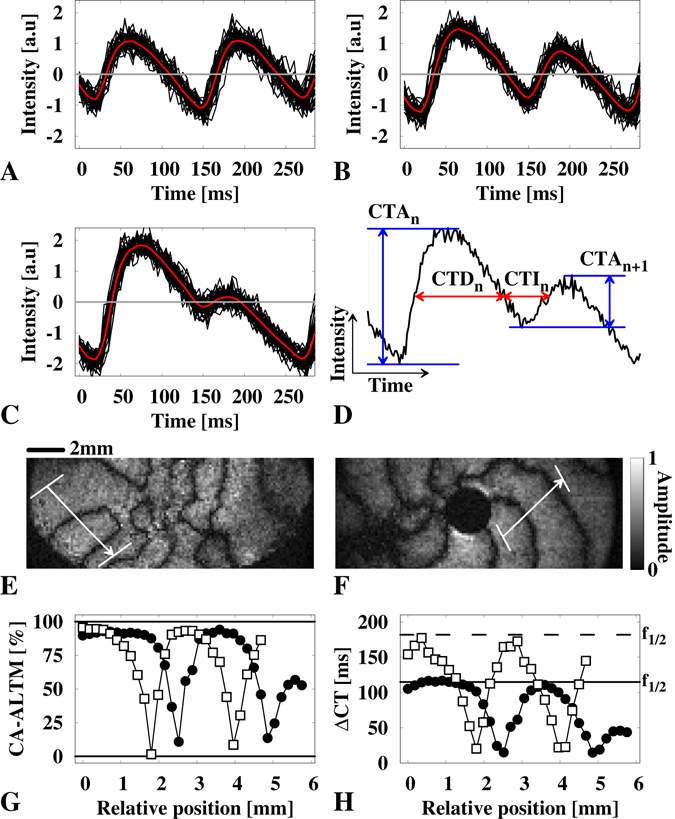
Analysis of spatially concordant alternans. (**A** to **C**) show three typical calcium signaling examples that are obtained using the signal oversampling technique (black lines). The average is shown as a red line that is used to quantify CA-ALTM and ΔCT (see Eqs (1) and (2)). A shows an example observed at a nodal line. (**B** and **C**) show examples of low (CA-ALTM ~ 100%) and strong (CA-ALTM ~ 50%) alternans, respectively. (**D**) shows an illustration of the cytosolic transient amplitude (CTA) and cytosolic transient duration/interval (CTD/CTI) in a typical single pixel recorded cytosolic calcium signal of high-frequency paced waves. CTD and CTI are determined by considering the intensity threshold at 50%. (**E** and **F**) show a free and an obstacle-anchored discordant alternating wave obtained by multidimensional Fourier analysis, respectively. The nodal lines are analyzed in line segments perpendicular to the nodal line orientation along a line segment of approximately 5 mm (see arrows in **E**) and (**F**). (**G** and **H**) show the CA-ALTM and ΔCT calculated along the line segment by Eq. (1) and by Eq. (2), respectively. The black circles and white squares correspond to the examples shown in E and F, respectively.

In experiments on the confluent cardiac tissues, we found that fluorescence signals from localized regions of a heart tissue exhibited oscillations with repetitive high-to-low and low-to-high peak-changes, which indicate the emergence of calcium alternans (Fig. 2A–C). To visualize spatial structures of alternans dynamics, we first applied a conventional method enabling the extraction of period-1 oscillations in cardiac tissues following the basic framework proposed by Uzelac and Fenton^34^. This image processing technique uses the periodic nature of cardiac wave activity to stack (average) steady state dynamics at equidistant time intervals and therefore reduce the noise ratio as the square root of the number of measurements. All oscillations are stacked in the case of period-1 oscillations, i.e., the intensity minimum of each oscillation is detected and sampled to one average representative oscillation, as illustrated by the black and red lines in Fig. 2A, respectively. For the application to SDA (period-2) dynamics, the time interval for analysis is chosen to be twice the oscillation period such that the long and short amplitudes align (Fig. 2B,C). This is essential for qualitatively extracting significant data of SDA and the dynamics of NL. The resulting average calcium signals (red lines, Fig. 2A–C) were used for the extraction of restitution properties, such as CTA and cytosolic transient duration/interval (CTD/CTI). A schematic illustration of CTA, CTD and CTI is presented in Fig. 2D. CTA depicts the calcium amplitude (|$(| C{{\rm{a}}}_{max}^{2+}\,-\,C{{\rm{a}}}_{min}^{2+}| )$) for each cytosolic oscillation and is more commonly used to describe cytosolic Ca^2+^ dynamics in cardiac cells. Conversely, CTD and CTI are obtained at an intensity level of 50% and is the equivalent description to the APD and DI of the membrane potential dynamics. It has been shown to be useful for the quantitative evaluation of the influence of drugs and calcium alternans in cardiac tissues^21, 44–46^. Although the method proposed by Uzelac and Fenton^34^ is powerful for visualizing local alternans dynamics, it does not provide detailed spatiotemporal information, particularly when the spatiotemporal evolution of NL and periodic-2 alternans changes are monitored. Therefore, knowing the exact position of NL at a certain time, as exemplarily indicated by dark stripes in Fig. 2E and F, particularly enables the extraction of precise spatiotemporal restitution dynamics.

### Quantification of spatiotemporal restitution dynamics

Two methods are commonly used to quantify intracellular calcium alternans, as illustrated in Fig. 2D. The magnitude of alternans (CA-ALTM)^40–43^ and the difference in the cycle length (Δ*CT*) of the long and short cytosolic transients similar to the extraction of APD and DI measurements for the membrane potential^45, 47, 48^. The calcium transient dynamics has been shown to be a better measure for estimating the bifurcation period in electrically entrained cardiac tissue grown on soft hydrogels^21^. Furthermore, it offers the advantage of extracting the local oscillation frequencies (Fig. 2H) compared to the CA−ALTM (Fig. 2G), which quantifies only the relative magnitude of the alternans amplitude.

**Figure 3:**
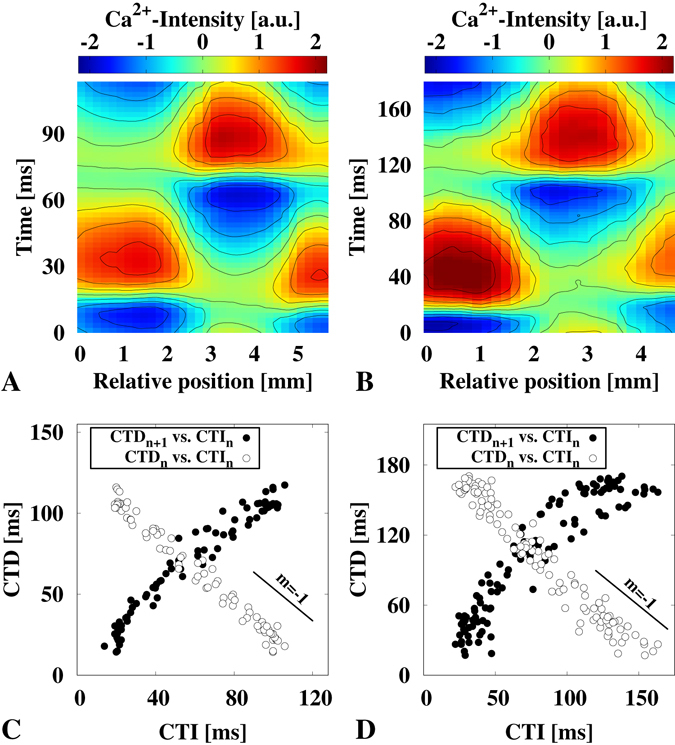
Spatiotemporal cobweb dynamics in concordant alternating spiral waves. (**A** and **B**) show the double period (*f*_1/2_) of the cytosolic calcium signaling along the line segments shown in Fig. 2A. The corresponding spatial cobweb dynamics are shown below in (**C** and **D**), respectively. The black circles indicate the restitution curve of the respective wave dynamics. The white circles highlight the slope of the intrinsic frequency *f* at which the spiral wave rotates around the wave singularity, and the obstacle, respectively, where *f* can be obtained as the interpolation to *f* ^−1^ = CTI(CTD = 0) or *f* ^−1^ = CTD(CTI = 0).

The extraction of the calcium transient dynamics in discordant alternans oscillating cardiac tissue opens the possibility of studying the restitution dynamics in greater detail. Cobweb dynamics are typically analyzed to quantify and visualize the APD restitution properties^48, 49^. Here we expand this framework to the spatiotemporal case of intracellular calcium dynamics. We assume that each spatial position (cellular patch) in the cardiac tissue exhibit the same electrophysiological properties. Integrating all selected spatial locations (Fig. 2E) to a single restitution map enables the precise visualization of the calcium transient dynamics (Fig. 3A and B), as well as a detailed mapping of the CTD restitution properties (Fig. 3C and D, black circles) using the cobweb scheme. The cross-section between the restitution curve (CTD_*n+1*_ vs. CTI_*n*_) with the envelop of a complete alternans cycle (white circles), e.g. long and short calcium transients (CTD_*n*_ vs. CTI_*n*_), corresponds to the positions where NL can be observed. The outward circles show alternans of different CA-ALTM depending on the distance from the cross section (for an illustrative experimental cobweb example, see the supplemental material, Fig. S2).

### Visualization of nodal lines

**Figure 4:**
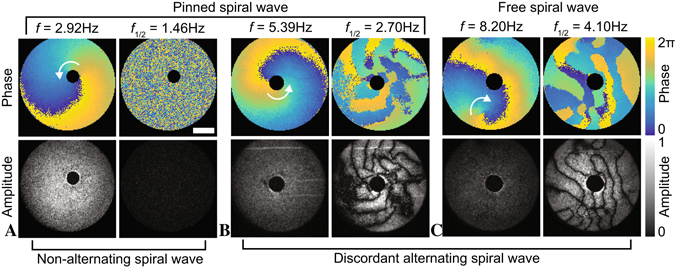
Experimentally observed discordantly alternating spiral waves in confluent cardiomyocyte tissue. The upper and lower images show the phase and amplitude of the spiral waves at the rotating frequency *f* (left) and the half-frequency *f*_1/2_ (right) in each panel, respectively. (**A**) shows a non-alternating (period-1) obstacle-anchored spiral wave, (**B**) shows an obstacle-anchored spiral wave, and (**C**) shows a free discordantly alternating (period-2) spiral wave. The amplitude is normalized for each spiral wave independently. The discordant alternating spirals show an increase in energy (amplitude) at *f*_1/2_ compared to the rotating frequency *f* of the spiral. Nodal lines (black regions) are detected at *f*_1/2_ as low-amplitude stripes that separate the high amplitude patches showing the spatially alternating wave dynamics.

NLs can be described as narrow bands of period-1 oscillation that spatially separate oscillations with higher periodicity. Similar dynamics in physics are classically analyzed using multidimensional Fourier transformation imaging, which is a powerful tool for revealing hidden information within the dynamic behaviors of an image series, i.e., by decomposing the original signal into oscillatory components (see Materials and methods). This method has been intensively used in experimental physics, i.e., on electromagnetic field propagations in antennas or periodic structures, thus revealing their spatially localized resonance frequencies^50, 51^. Furthermore, this method has been applied to ultra-high spatiotemporal resolution cardiac cell networks to visualize period-1 rotating spiral waves^52^, similar to that shown in Fig. 4A. However, we found that the full potential of Fourier transformation imaging is unleashed when applied to SDA wave dynamics in cardiac tissues to visualize NL (see Fig. 4B and C).

Stable spiral waves with rotation frequency *f* that exhibit period-2 SDA dynamics can be visualized by the spatial amplitude field at half the frequency of the spiral (*f*_1/2_) that corresponds to a pair of a small and a large Ca^2+^ oscillation, i.e. two spiral oscillations. In that case, the amplitude field is a measure for the resonance oscillation energy, which means the larger the differences in the peak intensity of the small and large Ca^2+^ oscillation, the higher the energy. Thus, visualizing the spatial amplitude field in tissue regions that exhibit period-2 oscillation show higher amplitude (energy) regions, and narrow bands of period-1 oscillation show lower amplitude (energy) regions (Fig. 4B and C). These bands are NL that spatially separate the anti-phase period-2 oscillations.

Thus, utilizing multidimensional Fourier analysis on cardiac tissue not only successfully uncovered the spatial structures of alternans dynamics with high spatial resolution (Fig. 4B and C) but also enables the investigation of local wave dynamics and properties by knowing the exact position of NL with greater precision (pixel-resolution).

### Analysis of nodal lines dynamics

**Figure 5:**
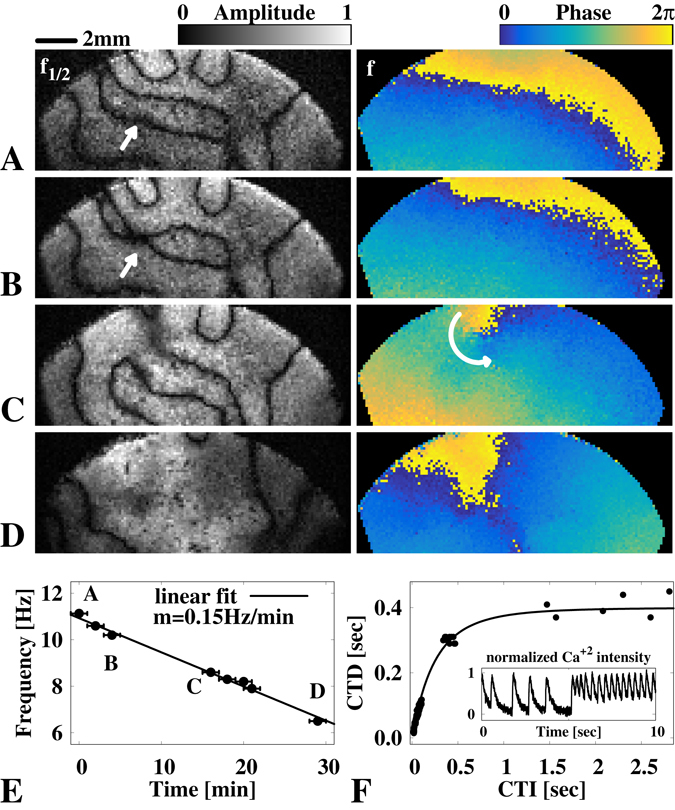
Spatiotemporal nodal line dynamics in a cardiomyocyte tissue. (**A** to **D**) show the amplitude (*f*_1/2_) and phase space (*f* ) of the spatiotemporal evolving nodal line dynamics of the free spiral wave (see Fig. 4C). A and B show the merging of a nodal line (white arrow). (**C**) shows the anisotropic merging after a nodal line break, which temporally induces a locally confined spiral wave (white curved arrow). (**D**) shows wave-curvature dominant dynamics induced by the linear decay of the spiral frequency *f*. The temporal change of *f* is shown in (**E**) within a time window of 30 minutes. (**F**) shows the estimated restitution curve of the cytosolic calcium dynamics extracted from A (CTD < 0.2 sec) and spontaneous wave activity (CTD > 0.2 sec) before the creation of the concordant alternans spiral wave. The latter is shown as an intensity time series in the inlet.

Next, we focus on the NL dynamics in cardiac tissue that exhibits discordant alternans through a single rotating spiral wave. We reviewed the farther region of the spiral tip (Fig. 4C upper half) and observed the dynamic evolution of the complex NL patterns over a period of approximately 30 min. Figure 5A to D show the amplitude and phase of the frequencies *f*_1/2_ (left side) and *f* (right side), respectively. The spatial phase information at *f* illustrates the wave propagation dynamics that correspond to the NL patterns in the amplitude field at *f*_1/2_. We observed a linear decay of the oscillation frequency, *f* (Fig. 5E), which indicates that the CTD restitution curve close to the critical point has a slightly lower slope than one, but is still linear considering the linear decay in *f*. Through this steady change in frequency we observed that the NL can drift, merge, annihilate and even form three-nodal complexes (Fig. 5A–D). Drifts in NL are observed up to an order of mm/min. This can lead to the merging of neighboring NL (see white arrows in Fig. 5A and B), where an even parity of NL crossings is conserved. Meanwhile, the wave dynamics can become locally unstable and form a spiral wave (see the amplitude and phase map on the white arrow in Fig. 5C), leading to an odd parity of the NL crossings. This odd parity is also observed at the tip of the spiral wave (Fig. 4C) and even consists of a multiple odd parity assembly of NL crossings. It could be argued that this enables the stability of the main spiral wave, whereas the spiral wave, as shown in Fig. 5C, is unstable and annihilates at the boundary of the tissue ensemble. Furthermore, we observe that with lower frequencies, the number of NL decreases. This result is probably due to the steady decay of the oscillation frequency, which also leads to an enhanced decrease of the CTD restitution slope. We reconstructed the entire CTD restitution curve (Fig. 5F) by assembling the extracted calcium transient dynamics of the initial alternans dynamics (Fig. 5A) and the spontaneous wave activities observed before the initialization of the spiral wave (Fig. 5F inset). Data shown at CTD <0.1 sec correspond to the alternans dynamics that are shown in Fig. 5A.

## Discussion

Our investigations showed that spatiotemporal patterns of NL can changes dynamically through the translation and merging of NL, leading to even and odd NL singularities. The latter is temporally unstable and associated with newly formed spirals that are locally confined. NL dynamics stabilizes macroscopic spiral waves, which exhibit changes in the rotation frequency. In other words, it can be stated that the spatiotemporal arrangement of out-of-phase alternating tissue patches stabilizes the synchronization of the global spatiotemporal wave formation in cardiac cell networks. This is not restricted only to spiral waves but would also apply to electrically entrained cardiac tissues. Our findings suggest that the changes in NL patterns are induced by changes in the spiral rotation frequency, since the slow (~mm/min) changes of NL patterns over time happen at the same rate as the observed changes in spiral frequency (~Hz/min). This result also implies that we observed electromechanically concordant alternans where calcium and APD alternans occur in phase^53^. This is because the SDA observed in this study is driven by the macroscopic spiral wave rotation with the frequency controlled solely by self-regulatory mechanisms, i.e., a balance of spatiotemporal membrane potential and intracellular calcium dynamics that are defined by the APD and conduction velocity restitution.

We combined an oversampling image processing technique with multidimensional Fourier analysis. The advantage of both techniques is that they are filter-free methods and thus do not exhibit signaling artifacts, as typically by conventional methods. Generating a series of Fourier images to extract the frequencies of the spatial tissue dynamics enables an analysis of patterns without the use of spatiotemporal filters or high-speed cameras. This in turn enables the use of very sensitive cameras that are required to extract the precise signal information needed in the medical sciences. In this study, we were able to extract SDA by choosing line segments that are parallel to the NL, whereas an automatic scan of a 4 × 4 ROI does not lead to a successful analysis due to overlap of period-1 and period-2 dynamics in the ROI. Conversely, line segments would lead to even worse results because the exact NL positions would not be known. Therefore, our newly introduced methodology opens up new possibilities to study the pattern formation of SDA^54^, localizing tissue heterogeneity^55^ and controlling the spatiotemporal dynamics in macroscopic NL patterns.

In this study, we have discussed period-2 alternans (*f*_1/2_, 2: 2 type of alternans rhythm); however, the proposed method is also applicable to other more complex Wenckebach-like calcium rhythms, i.e., 4: 3 and 3: 2 that can be observed in electrically entrained cardiac tissues^54^. This can be further improved by considering time-domain Fourier analysis to take temporal variations of alternans into account. Currently available fast, high-resolution microscopy and Fourier imaging feedback controlled stimulation protocols may enable the active control of SDA patterns in cardiac tissues, thus enabling new insights to be obtained in the understanding of life-threatening cardiac diseases^56^, and other biological and non-biological excitable systems, such as engineered bioelectric tissue^57^, neuronal networks^6^ and Belousov-Zhabotinsky reaction-diffusion systems^58, 59^.

## Electronic supplementary material

Supplementary information
