# Supplementary material for: Dynamics of spatiotemporal line defects and chaos control in complex excitable systems
Source: Sci Rep. 2017 Aug 10;7:7757. doi: 10.1038/s41598-017-08011-z (PMC5552747; doi:10.1038/s41598-017-08011-z)
Supplement: Supplementary file 1 — Supplementary information [file 41598_2017_8011_MOESM1_ESM.pdf]

Supplementary Figures

Dynamics of spatiotemporal line defects and chaos control in complex excitable systems.

Marcel Hörning, François Blanchard, Akihiro Isomura, Kenichi Yoshikawa

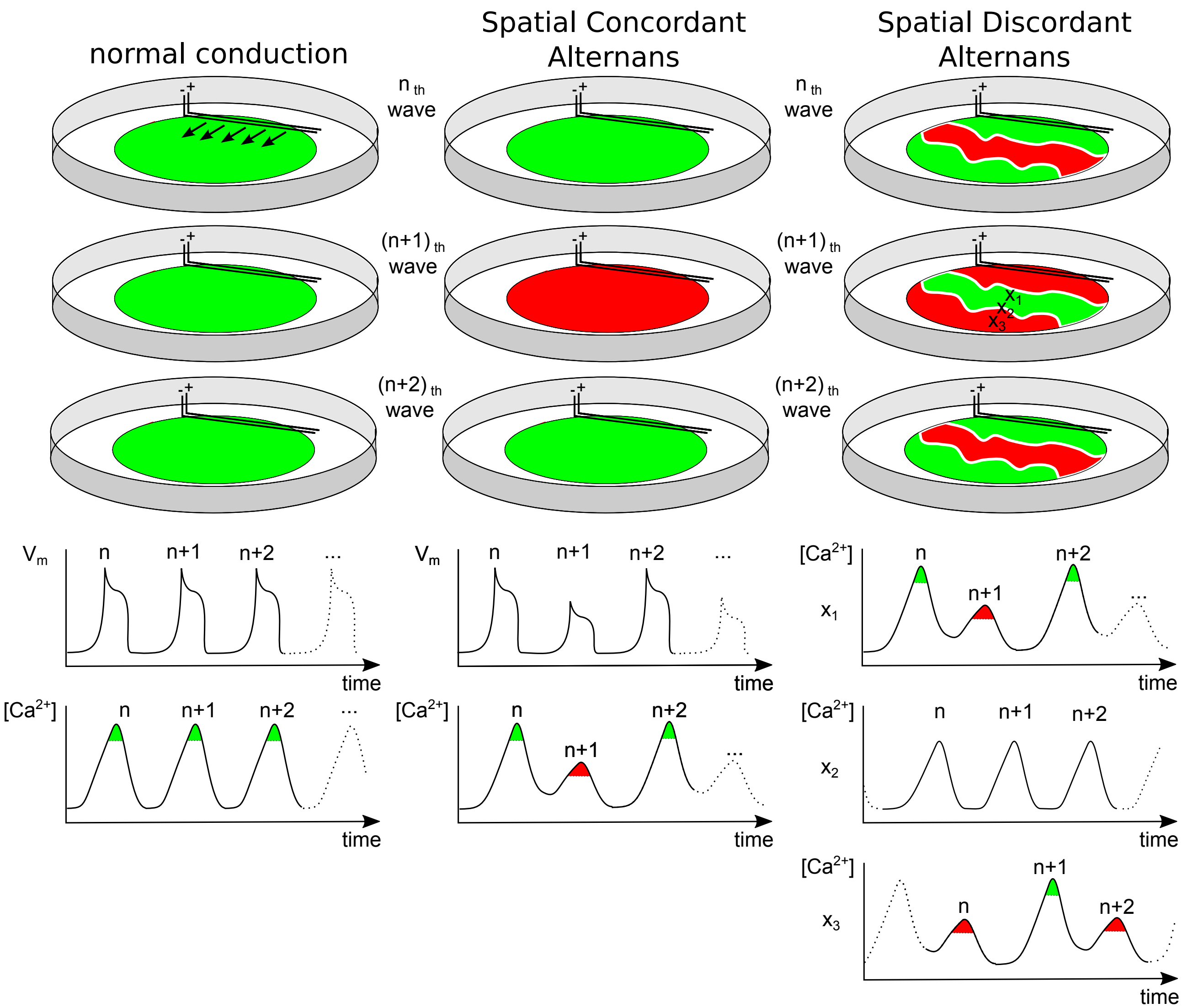

Figure S 1. Schematic illustrations of normal wave conduction (left), spatial concordant alternans (center) and spatial discordant alternans (right) in high-frequency entrained cardiac tissue. The respective membrane potential (V_m) and intracellular Calcium ($[Ca^{2+}]$) dynamics are illustrated below. Spatial concordant alternans is illustrated as electromechanical concordant alternans, where the membrane potential V_m and calcium transients $[Ca^{2+}]$ occur locally in phase.

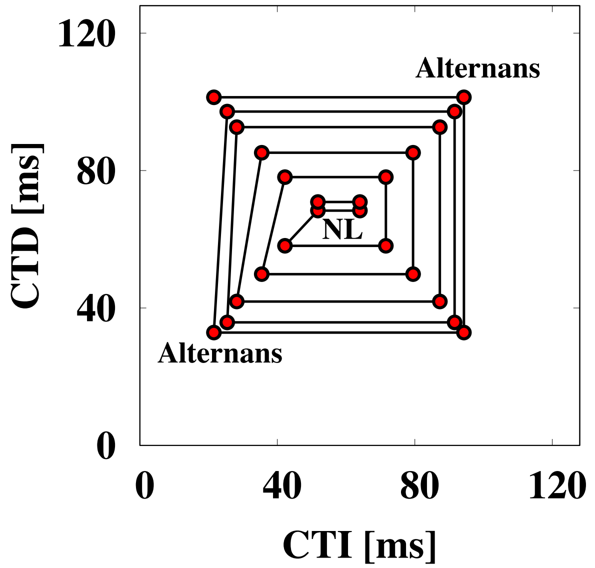

Figure S 2. Visualization of the cobweb in a calcium transient restitution curve that shows a spatial transition from a local period-2 alternans oscillation site (see 'Alternans', CA-ALTM∼100%) to a period-1 NL oscillation site (see 'NL', CA-ALTM∼0%) in six equidistant spatial steps of 180μm. (see Fig. 2E)

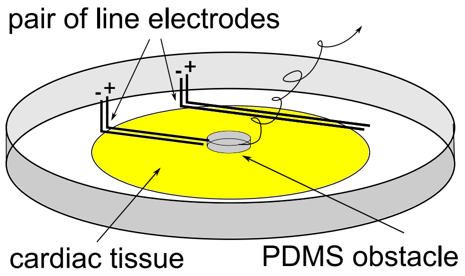

Figure S 3. Spatial arrangement of the experimental setup to induce a spiral wave in a cardiac tissue. The PDMS obstacle is removed after 24 hours of cell seeding. The electrodes are triggered with a delay to induce a rotating spiral wave in the cardiac tissue.
